# ST elevation revealing acute myocarditis with SARS cov 2 infection: Case report

**DOI:** 10.1016/j.amsu.2021.102313

**Published:** 2021-04-17

**Authors:** S. Taouihar, A. Bouabdallaoui, M. Aabdi, G. Elaidouni, H. Bkiyar, N. Aichouni, N. Smaili, N. ElOuafi, I. Skiker, B. Housni

**Affiliations:** a Anesthesiology and Intensive Care Unit, Mohammed VI University Hospital, University MOHAMMED I, Oujda, Morocco; b Cardiology Department, Mohammed VI University Hospital, University MOHAMMED I, Oujda, Morocco; c Radiology Department, Mohammed VI University Hospital, University MOHAMMED I, Oujda, Morocco; d Simulation Center for Medical Formation, University MOHAMMED I, Oujda, Morocco

**Keywords:** Covid-19, Myocarditis, ST elevation

## Abstract

**Introduction:**

SARS-CoV-2 viral infection can manifested by respiratory symptoms, or other symptoms, such as the cardiovascular manifestations including acute coronary syndrome, pericardial effusion, and heart failure.

**Clinical case:**

A 51-year-old patient admitted to the emergency room for epigastric pain with no respiratory signs and with an ST-segment elevation inelectrocardiogram that ultimately revealed myocarditis and SARS CoV-2 2 infection.

**Conclusion:**

The clinical manifestations of SARS CoV-2 might be atypical, and the diagnosis might be considered in this pandemic area

## Highlights

• SARS-CoV2 manifests usually with respiratory symptoms, but it can manifests with others signs including cardiovascular like myocarditis.
• In this case, the patient represent with ST elevation and myocarditis, a rare revelation mode.

## 1 Introduction

Covid-19 disease caused by Severe Acute Respiratory Coronavirus-2 (SARS-CoV-2) affects primarily the respiratory system and it is usually manifested by respiratory symptoms, but also affects other systems such as the cardiovascular system, which may be the first warning signs of sars-cov-2 infection like acute coronary syndrome, pericardial effusion, and heart failure [1].

In this paper, we will represent a case of a 51 years old patient admitted to the emergency room for epigastric pain and the explorations revealed acute myocarditis with Sars cov 2 infection.

The importance of the case: acute myocarditis is a rare revealing diagnosis of Sars cov 2-infection diagnosis.

## 2 Case report

A 51 years old patient with medical history of hyperthyroidism on carbimazol was admitted to the emergency room with intense epigastric pain at rest and effort, associated with nausea and vomiting progressing for 5 days.

The initial physical clinical examination was as follow: blood pressure at 123/60 mmhg, tachycardia with 130 B/min, respiratory rate at 19 breathts/min, with pulse oxymetry at 70% on ambient air, temperature at 37.2 °C, with bilateral rhonchis, the rest of the examination was normal.

**Fig. 1:**
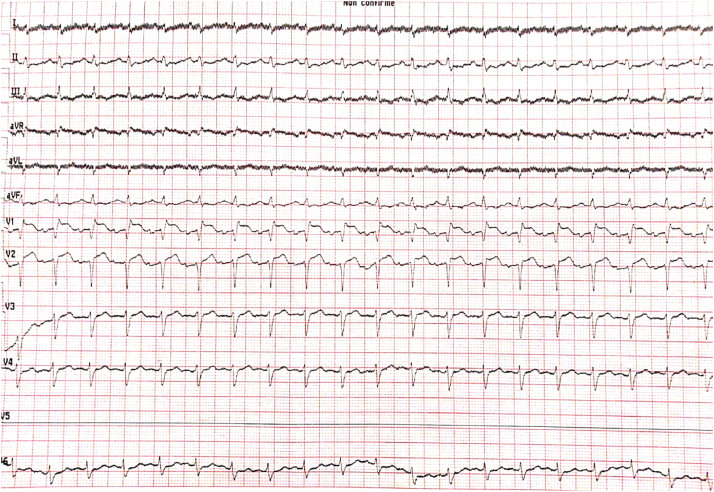
ECG showing an ST segment elevation in V1 and V2 derivations with Q waves of necrosis, an abrasion of R waves in V3 and a negative T wave in inferiorterritory.

The patient was admitted to intensive care unit and put on hight concentration mask with improvement of the saturation 90% under 8l/min, the electrocardiogram (ECG) found a ST segment elevation in V1 and V2 derivations with Q waves of necrosis, an abrasion of R waves in V3 and a negative T wave in inferior territory (Fig. 1).

The biological assessment showed a very high ultra-sensitive troponin 20.000 ng/l (1000 times higher than normal) , white lymphopenia at 850/mm3 blood cells at 14.000/μl, C-reactive protein at 120 mg/l (normal between 0.00 and 5.00 mg/l), ferritin at 500 μg/, D-dimer at 0.40, lipasemia at 58 IU/l (normal 8.00–78.00) IU/l.

The transthoracic echocardiogram (TTE) showed akinesia of the tip of the antero-septal and inferior wall of the left ventricular (LV) with severe hypokinesia of the inferior wall, a systolic dysfunctionof LV with an ejection fraction of 40%.

The initial therapeutic management was aspirin 300 mg, clopidogrel300 mg and simvastatin 20mg, proton pump inibitor PPI 40mg.

**Fig. 2:**
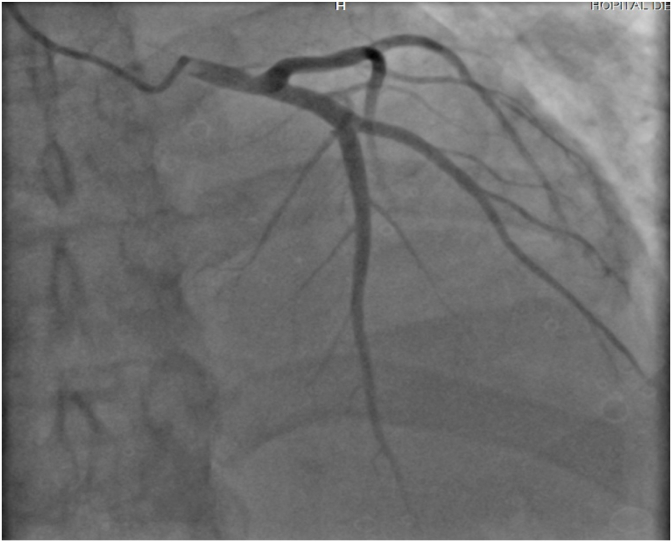
Coronary angiography showing a Normal left coronary arteries.

**Fig. 3:**
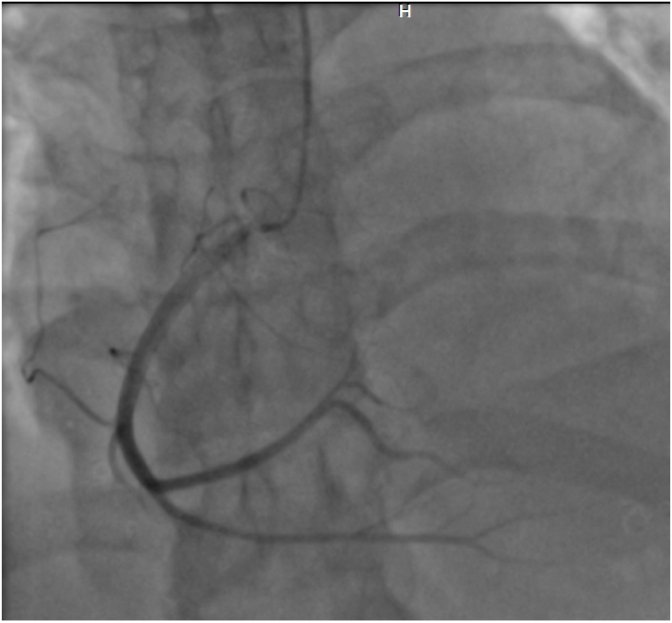
Coronary angiography showing Normal right coronary arteries.

An emergency coronary angiography performed was normal (Fig. 2 and Fig. 3).

**Fig. 4:**
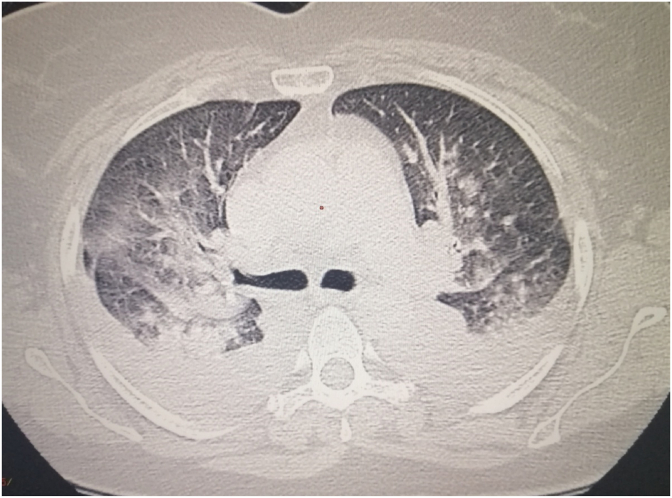
Thoracic CT scan showing 50% of lung damage.

Given the respiratory distress, a thoracic computed tomography CT scan was performed showing aspect of covid 19 pneumopathy with 50% of lung damage and bilateral pleural effusion (Fig. 4).

The polymerase chain reaction (PCR) for SARS COV 2 infection was positive.

**Fig. 5:**
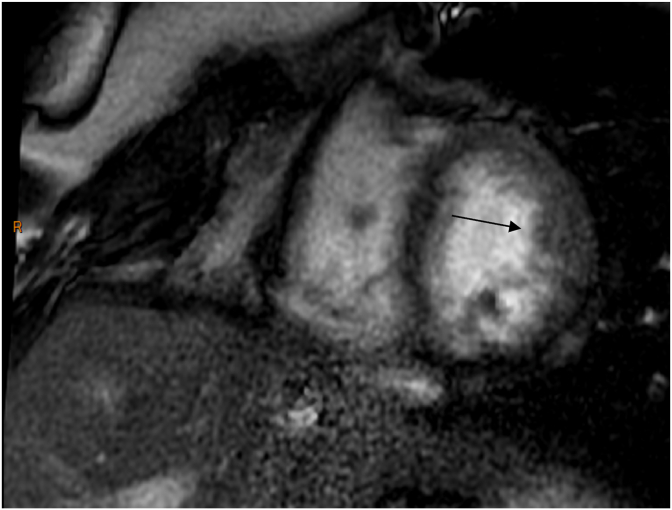
FIESTA MRI sequence: Cardiac short-axis slices showing left ventricle myocardial thickness

**Fig. 6:**
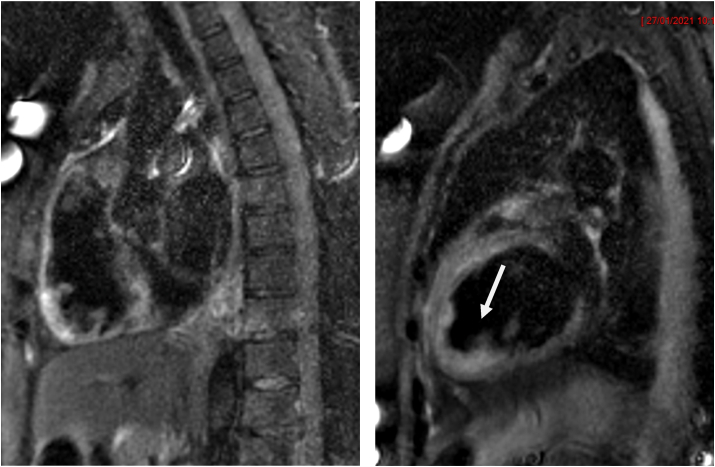
T1-weighted sagittal sequence (10 minutes after injection of contrast product): focal thickening (arrow), with an exaggerated enhancement compared to the rest of the myocardium.

Given the electrocardiographic, echocardiographic, biological and coronary angiography data, a magnetic resonance imaging (MRI) was performed showing a focal hypertrophy in the antero-septal and inferior segment of the LV extended over approximately 62mm (Fig. 5), and in antero-inferior of the RV extended over 27mm, in hyper signal T2, with an intense enhancement on the tardive injected sequences compared to the rest of the myocardium in favor of a myocarditis (Fig. 6).

The diagnosis of sars cov 2 infection revealed by acute of myocarditis was maintained and the patient was put on plavix kardegic, converting enzyme inhibitor, bisoprolol, and sars cov 2 infection treatment: vitamin c, zinc, azythromicin, dexamethasone and curative anticoagulation Enoxaparin.

The evolution was favorable on clinical and biological levels, with a pulse oxymetry of 93% on ambient air on the 3rd day with a decrease of troponin numbers.

The patient was discharged on day 7.

## 3 Discussion

Sarscov 2 viral infection is a single-stranded RNA virus invades myocardial, lung and kidney cells by attaching to the ACE2 receptor expressed on their surfaces.

Although the clinical manifestation is dominated by pulmonary manifestations, cardiovascular manifestations have been described such as acute coronary syndromes and myocarditis whose clinical presentation is very varied ranging from simple atypical chest pain to cardiogenic shock.

BAVISHI and al reported 20% of cardiac manifestations in 26 studies including 11,685 patients infected by SARS-cov 2 virus [2].

Acute coronary syndrome is a frequent condition presenting with unstable angina, myocardial infarction without elevation of ST-segment and myocardial infarction with elevation of ST-segment [3]. The latter grouping together two entities, one with obstruction of the coronaries and the other without obstruction or with a stenosis less than 50% of the coronary arteries in coronographycalled Myocardial Infarction with Normal or Non- Obstructive Coronary Arteries (MINOCA) whose etiologies are multiple ranging from coronary causes (such as coronary spasms or rupture of plaque, etc.) to non-coronary causes, particularly myocarditis, inflammatory diseases such as taku-tsubu and pulmonary embolism [4].

In our case, based on the clinical, electrocardiographic, biological data and with a normal coronarography, we completed by a cardiac MRI suspecting an acute viral myocarditis.

In covid positive patients, the prevalence of myocarditis remains unknown.

Jamie SY Ho and al described the case of 51 patient infected with Sarscov 2, 12 cases had a confirmed myocarditis, however 39 cases were diagnosed with probable myocarditis [5].

In another study involving 84 infected patients 4.8% had viral myocarditis. [6].

Myocarditis is defined by an inflammatory infiltrate in the myocardium associated with areas of non-ischemic necrosis [7], whose etiology may be infectious, toxic, autoimmune or other.

The clinical diagnosis is based on multiple arguments: [8].

• Polymorphic clinical presentation
• Electrocardiogram: repolarization disorder, ventricular rhythm disorder and conduction disorders
• Biological: elevation of serum troponin level, CRP, VS, eosinophilia, and hyperleukocytosis
• Echocardiography showing hypertrophy of the ventricular walls, impaired cardiac function
• Magnetic resonance imaging which represents the gold standard in the diagnosis, management and follow-up of the disease.

The diagnosis of myocarditis is based on the presence of two of the following three Lake Louise criteria [9] (08):

➢ Myocardial hyperemia, demonstrated by early global enhancement of the myocardium with gadolinium in a T1-weighted sequence;
➢ Regional or global myocardial edema, demonstrated by hyper signal in T2-weighted sequence;
➢ Myocardial necrosis or fibrosis most often multifocal epicardial localization (as opposed to endocardial scars of ischemic origin), demonstrated by late enhancement with gadolinium in T1-weighted sequence.

The treatment of acute myocarditis is based on two components, treatment of heart failure and etiological treatment (antiviral, beta interferon, corticosteroid therapy, and immunosuppressant) (08).

## 4 Conclusion

Sarscov 2 infection manifest mainly with respiratory symptoms, but can also manifest with cardiac involvement which can be severe and one with a non-negligible mortality rate.

The diagnosis of myocarditis revealed by an acute coronary syndrome ST positiveremains uncommon and requires careful investigation based on a range of clinical and paraclinical arguments.

The case report follows care 2018 guidelines [10].

## Ethical approval

The ethical committee approval was not required given the arti-cle type (case report).

## Consent

Written informed consent was obtained from the patient.

## Sources of funding

This research did not receive any specific grant from funding agencies in the public, commercial, or not-for-profit sectors.

## Author contribution

Taouihar Salma: study concept, Data collection; data analysis; writing review & editing, Bouabdellaoui amine: Study conception, data analysis. AABDI Mohammed: contributor., Elaidouni Ghizlane: contributor., BKIYAR Houssam: contributor., Aichouni narjiss: contributor., Smaili Nabila: Supervision and data validation., Elouafi Nouha: Supervision and data validation., Skiker Imane Supervision and data validation, HOUSNI Brahim: supervision and data validation research registration (for case reports detailing a new surgical technique or new equipment/technology)

## Trial registry number

As this manuscript was a case report with no new medical device nor surgical techniques, not prior registration is required.

## Consent

Written informed consent was obtained from the patient for publication of this case report and accompanying images.

## Guarantor

Taouihar Salma; Bouabdellaoui Amine.

## Provenance and peer review

Not commissioned, eternally peer reviewed.

## Declaration of competing interest

The authors state that they have no conflicts of interest for this report.
